# Vitamin D, Cellular Senescence and Chronic Kidney Diseases: What Is Missing in the Equation?

**DOI:** 10.3390/nu15061349

**Published:** 2023-03-10

**Authors:** Romina P. Martinelli, Sandra Rayego-Mateos, Matilde Alique, Laura Márquez-Expósito, Lucia Tejedor-Santamaria, Alberto Ortiz, Emilio González-Parra, Marta Ruiz-Ortega

**Affiliations:** 1 Cellular Biology in Renal Diseases Laboratory, IIS-Fundación Jiménez Díaz-Universidad Autónoma, 28040 Madrid, Spain; 2 Ricors2040, 28029 Madrid, Spain; 3 Departamento de Biología de Sistemas, Universidad de Alcalá, Alcalá de Henares, 28871 Madrid, Spain; 4 Instituto Ramón y Cajal de Investigación Sanitaria (IRYCIS), 28034 Madrid, Spain; 5 Department of Nephrology and Hypertension, IIS-Fundación Jiménez Díaz-Universidad Autónoma Madrid, 28040 Madrid, Spain

**Keywords:** vitamin D, cellular senescence, biological aging, premature aging, chronic kidney diseases

## Abstract

As life expectancy increases in many countries, the prevalence of age-related diseases also rises. Among these conditions, chronic kidney disease is predicted to become the second cause of death in some countries before the end of the century. An important problem with kidney diseases is the lack of biomarkers to detect early damage or to predict the progression to renal failure. In addition, current treatments only retard kidney disease progression, and better tools are needed. Preclinical research has shown the involvement of the activation of cellular senescence-related mechanisms in natural aging and kidney injury. Intensive research is searching for novel treatments for kidney diseases as well as for anti-aging therapies. In this sense, many experimental shreds of evidence support that treatment with vitamin D or its analogs can exert pleiotropic protective effects in kidney injury. Moreover, vitamin D deficiency has been described in patients with kidney diseases. Here, we review recent evidence about the relationship between vitamin D and kidney diseases, explaining the underlying mechanisms of the effect of vitamin D actions, with particular attention to the modulation of cellular senescence mechanisms.

## 1. Introduction

The last decades have witnessed a dramatic increase in global life expectancy. The World Health Organization (WHO) estimates that by 2030, one in six people will be aged over 60 years, and by 2050, this will rise to 22%. Unfortunately, this boost in the human lifespan is associated with a rise in age-related diseases, such as type 2 diabetes mellitus, Alzheimer’s disease, cardiovascular diseases and chronic kidney disease (CKD). Among these diseases, CKD is one of the fastest-growing global causes of death and it is expected to become the fifth most common cause of death worldwide by 2040, being increased at the end of the century to the second position [1]. CKD is a common, progressive and irreversible disorder [2]. Lifestyle and drug interventions (e.g., SGLT2 inhibitors and ACE inhibition or angiotensin II receptor type 2 blockade) are currently the cornerstone for treatment for CKD. Still, they do not sufficiently prevent the progression of the disease. Thus, there is a high demand for better treatment options that stop CKD progression and even reverse CKD. In addition, there is a lack of treatments for acute kidney injury (AKI) that may prevent or delay the development of CKD [3,4,5]. Furthermore, CKD remains underdiagnosed and undertreated, and biomarkers are needed for earlier detection of kidney damage or for better prediction of progression [3,4,5]. Finally, CKD is characterized by premature aging in the skeletal, immune and cardiovascular systems. Even children with kidney diseases develop atherosclerosis, showing a bidirectional aging–CKD relationship [1], suggesting that CKD can be considered as a clinical model of premature aging.

Aging is a natural and complex process resulting in impaired physiological functions in all cells, tissues and finally organs, resulting in homeostasis loss. The hallmarks of the aging phenotype include genomic instability, telomere shortening, epigenetic changes, loss of proteostasis, dysregulation of the nutrient sensor pathways, mitochondrial dysfunction, stem cell exhaustion, altered intercellular communication, chronic inflammation, dysbiosis and senescence [6,7]. These changes also happen in age-related diseases that share similar pathogenic processes, as described in CKD. Nowadays, intensive research is focused on deciphering the mechanisms of aging and finding new therapeutic strategies to treat age-related disorders and increase lifespan [8,9]. Interestingly, some of these therapeutic options can also ameliorate renal damage, as we discuss below.

The vitamin D endocrine system is an essential regulator of various physiological functions in the human body. Apart from its role in maintaining calcium balance and bone health, it has been found to possess numerous non-skeletal effects. Many immune cells, including dendritic cells, macrophages, and T and B cells, can produce vitamin D and express the vitamin D receptor, showing the interrelation of vitamin D and the immune system [10,11]. Recent preclinical studies have highlighted the beneficial effects of treatment with vitamin D or its analogs in inflammatory diseases by exerting anti-inflammatory effects, including its ability to modulate the expression of genes involved in the regulation of the immune system or pro-inflammatory factors [12,13]. The kidneys are the primary regulators of the endocrine vitamin D system, playing a key role in regulating systemic, active vitamin D levels. The tubular epithelial cells are responsible for the production of the active form of vitamin D, 1,25(OH)2VD3 or calcitriol [14,15,16,17,18,19,20]. In patients with CKD, there is a gradual decline in the kidney’s ability to produce 1α-hydroxylase, resulting in a decrease in the activation of vitamin D. This results in the lowering of vitamin D circulating levels, which can lead to various health complications. Vitamin D deficiency has been linked to several diseases, including cancer, Alzheimer’s disease, type 2 diabetes mellitus, cardiovascular disease, autoimmune diseases and aging [14,15,16,17,18,19,20].

Here, we review recent evidence on the relationship between vitamin D, CKD and aging. In addition, the emerging evidence on the pleiotropic effects of treatment with vitamin D or its analogs in CKD is also reviewed, describing the underlying mechanisms elicited by vitamin D, with special attention to the modulation of the cellular senescence mechanisms.

## 2. Cellular Senescence in Biological Aging and Age-Related Disorders

During the natural biological process of aging, both extrinsic and intrinsic agents can cause accumulative damage in the cells, leading to the activation of cellular senescence-related mechanisms [21]. Cellular senescence is a process where cells decrease their proliferative capacity and are no longer being able to divide, in association with unique characteristics, such as flattened and enlarged morphology, increased expression of cell cycle inhibitors and elevated senescence-associated β-galactosidase activity [22]. Notably, the excessive accumulation of senescent cells in different organs and biological systems leads to a functional decline [6,7]. The senescence phenotype is characterized by an aberrant secretome known as the senescence-associated secretory phenotype (SASP). The components of the SASP include pro-inflammatory cytokines, profibrotic factors and extracellular vesicles [23,24,25]. These factors released by senescent cells can act on the surrounding cells inducing secondary senescence [22], therefore contributing to the amplification and progression of the damage. Further research in this area offers a chance to design new therapeutic approaches, including inhibitors of the cell cycle or SASP components, as well as eliminating senescent cells.

During biological and premature aging, senescence-related mechanisms activate in all types of the cells of the entire organism. These implicate significant consequences for the immune system, inducing two major events. First, there is a low-grade inflammation, known as inflammaging [26], and second there is a decrease in innate and adaptative functions, called immunosenescence [27,28]. This highlights the relevance of the deepening understanding of the relation between immune cells and aging. Nevertheless, senescence is necessary for correctly maintaining tissues, organs and physiological processes, such as embryogenesis, development, and wound healing [22,29,30]. Moreover, molecular and cellular senescence-related mechanisms can be also activated in response to injury and are involved both in maladaptive responses and the endogenous repair mechanisms, as described in kidney injury [2]. The complexity of these mechanisms emphasizes the importance of future research in this area.

### Senescence: The Molecular View

Cellular senescence is a hallmark of aging. This process can be triggered by different types of damage, including oncogene activation, the DNA damage response (DDR) pathway [31], telomere shortening, mitochondrial injury, viral or bacterial infection, reactive oxygen species (ROS) production, nutrient imbalance, and mechanical stress [22,32]. Senescent cells are characterized by a permanent cell cycle arrest in the G1 or G2 phases, mediated by the activation of the tumor suppressor protein p53, the inhibitors of the cyclin-dependent kinases CDKN2A/p16 and CDKN1A/p21, as well as the retinoblastoma-1 (RB1) family proteins [21]. Cellular senescence activation provokes several intracellular metabolic and functional changes, including mitochondria dysfunction and redox disbalance together with transcriptional reprogramming, leading to a phenotype change. As commented before, one key feature of senescent cells is a specific secretome, the SASP. Many components of the SASP are regulated by the transcription factors NF-κB and C/EBP (CCAT/Enhancer Binding Protein) [33,34,35], suggesting that targeting the activation of these transcription factors could be used as anti-aging strategies. The SASP comprises a core of growth factors, cytokines and chemokines, but its composition depends on the cell type and the trigger stimulus. Although senescence transcriptome signatures have been identified [36], a recent study based on proteomics showed a lack of correlation between transcriptome and proteome [37]. Nevertheless, the authors identified a top core SASP, constituted by GDF15, STC1, SERPINs and MMP1, proteins that can be used as aging plasma markers [36]. Future studies are needed to identify further and define senescent markers, which can differ depending on the tissue and pathological condition.

## 3. CKD and Cellular Senescence

Recent preclinical research has shown the important role of cellular and molecular senescence-related mechanisms in natural aging, AKI and in the AKI-to-CKD transition [2,38,39,40,41]. In C57BL/6 mice, natural aging was characterized by decreased Klotho gene expression and activation of DDR in the kidneys as soon as 12 months. Moreover, prolonged DDR and permanent cell cycle arrest associated with inflammatory cell infiltration and further Klotho downregulation was noted in the kidneys of 18-month-old mice. These findings show early senescence-related changes in the kidney preceding renal dysfunction, which was observed in 18-month-old mice [39]. The regulation of the redox balance has been proposed as a key mechanism involved in the early activation of cellular senescence in renal injury. ROS production and kidney inflammatory cell infiltration are triggered by acute tubular damage induced by a nephrotoxic drug or ischemia–reperfusion injury (IRI). As parts of the damaged tubular epithelial cells fail to undergo efficient DNA repair or apoptosis, they become senescent at a premature age (3 months), similar to the observation in naturally aging mice [40,41]. However, when aged mice are subjected to AKI, all these processes and mechanisms are magnified [41]. Moreover, in naturally aging mice, the early senescence activation observed at 12 months was associated with redox imbalance, deregulation of nuclear factor (erythroid-derived 2)-like 2 (Nrf2), increased lipid peroxidation and kidney inflammation [39]. Nrf2 is the master regulator of inducible antioxidant responses that can attenuate cellular injury from oxidative stress [42]. Preclinical studies in different AKI models have described early cellular senescence activation, including upregulation of p21 expression. Moreover, treatment with a p53 inhibitor in IRI has demonstrated the importance of G1 cell cycle arrest in the AKI-to-CKD transition and progression of fibrosis [38,39,40,41]. Future studies are still needed to unravel the cellular and molecular mechanisms involved in kidney injury and repair, including the failure of the repair process.

Interestingly, the controlled elimination of senescent cells (“senotherapy”) extends a healthy lifespan and is associated with preserved kidney function [43]. Another potential mechanism implicated in kidney damage can be due to the secondary senescence caused by the induction of SASP by injured tubular cells. Recent studies targeting SASP components have shown renal protective effects. In this sense, IRI studies in CCN2-deleted mice showed that CCN2 is a part of the SASP secretome that could also trigger senescence by itself. In cultured murine tubular epithelial cells, stimulation with CCN2 induced a senescent cellular phenotype [38]. In addition, human kidney grafts showed increased CCA-related proteins associated with the upregulation of CCN2 protein, confirming the clinical relevance of murine studies [38]. However, additional preclinical studies are necessary to evaluate the extent to which targeting senescence in CKD or kidney grafts decreases fibrosis and improves kidney function and graft survival.

## 4. Vitamin D, Aging and CKD

Vitamin D pathway defects can disturb the kidney’s ability to activate this hormone [44]. Moreover, low levels of vitamin D have been observed in conditions such as premature aging and CKD, which could have implications for disease progression and patient outcomes. Despite this evidence, many unanswered questions in this field still require further investigation to fully understand the impact of vitamin D deficiency and defects in the vitamin D pathway on human health. Here, we briefly review the main mechanisms of vitamin D action, and its relationship with aging, immune system regulation and kidney injury.

### 4.1. Vitamin D Synthesis and Mechanisms of Action

The main circulating form of vitamin D, 25-dihydroxy vitamin D3 (25(OH)VD3), is synthesized in the liver from vitamin D3 (VD3), which is a result of the conversion of 7-dehydrocholesterol in the skin. VD3 can also be acquired from the diet. The enzyme 25-dihydroxyvitamin D3–1α-hydroxylase catalyzes its transformation to 1,25(OH)2VD3, also called calcitriol or 1,25D, the most active native vitamin D metabolite and a potent steroid hormone [45]. This enzyme is expressed in large amounts by mitochondria in kidney proximal tubular cells, but it can also be found in various tissues, including the immune system. Therefore, many cell types can locally produce 1,25D, and it can display autocrine or paracrine functions, explaining its pleiotropic properties [46]. On the other hand, 1,25D is inactivated mainly in the kidney and intestine by the enzyme vitamin D 24-hydroxylase [47]. Vitamin D deficiency is related to insufficient sun exposure and is frequent in winter and in the elderly, as well as in several diseases [14,15,16,17,18,19,20]. Interestingly, vitamin D supplementation should be considered whenever there is insufficiency or deficiency of vitamin D, i.e., when serum 25-OH-vitamin D levels are below 30 ng/mL [48].

Vitamin D exerts its functions through the vitamin D receptor (VDR) [44]. VDR is a member of the nuclear receptor superfamily and upon 1,25D binding, it behaves as a transcription factor of specific genes classified as primary and secondary target genes [49,50]. Primary target genes are mainly composed of transcription factors directly regulated by 1,25(OH)2VD3-activated VDR. They control the expression of secondary target genes, which are predominantly downregulated: some of the anti-inflammatory actions of vitamin D are modulated in this manner [51]. Hence, 1,25D binding to VDR regulates the transcription of genes that control mineral homeostasis and skeletal health, but also genes that regulate immune, kidney and cardiovascular functions [43]. VDR is expressed in many cell types, therefore the ubiquitous expression of VDR can explain the wide range of actions of vitamin D besides its classical actions on calcium metabolism [52], including hematopoietic stem cell production, immune system regulation, cell cycling and different metabolic functions [53,54,55].

Vitamin D can also activate other membrane receptors, such as the steroid-binding protein 1,25D(3)-MARRS, also named ERp57/GRp58 [56,57]. This receptor mediates non-genomic actions associated with rapid membrane-initiated signaling [57]. In intestinal cells, 1,25D3-MARRS mediated calcium [58,59] and phosphate [60] absorption by rapidly stimulating their transport. The role of 1,25D3-MARRS has been most extensively studied in cancer [61,62,63], which is associated with extracellular and intracellular redox activities and the sequestration of STAT3 [64]. For the kidney, the data are scarce. Increased ERp57 urine levels were observed in the early stages of human CKD patients and correlated with the severity of renal fibrosis [65], as observed in experimental renal fibrosis [65]. However, in vitro studies performed in cultured tubular epithelial cells showed that the anti-inflammatory actions of the VDR agonist paricalcitol, including NF-κB2 activation, were not mediated by MARRS/ERp57 [66]. These findings suggest that more studies are needed to define the role of 1,25D3-MARRS in vitamin D actions in the kidney.

### 4.2. Vitamin D as an Anti-Aging Factor

**Figure 1:**
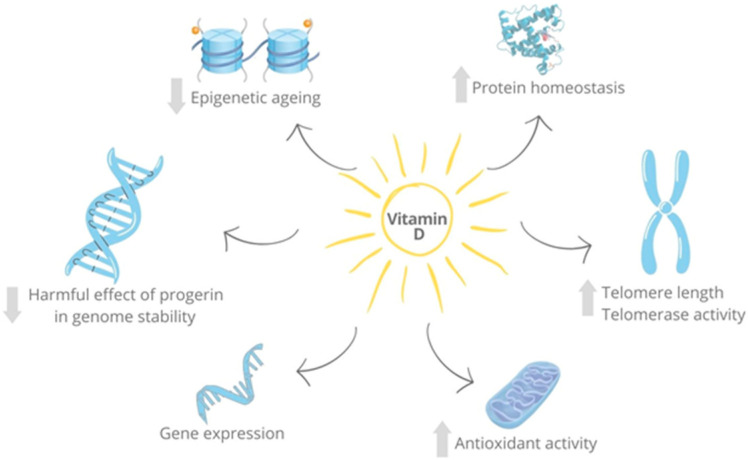
Pleiotropic actions of vitamin D related to cellular senescence. 1,25D counteracts the effects of progerin on DNA stability, helps to regulate epigenetic age, maintains protein homeostasis, preserves telomere length, regulates gene transcription and reduces oxidative stress by regulating pro-oxidant and antioxidant genes.

Some non-classical actions of vitamin D may counteract the hallmarks of aging [6,7] (Figure 1). In vitro experiments have shown that 1,25D reduces the harmful effect of progerin on genome stability. Progerin is a mutant form of the LMNA gene expressed in progeria syndrome that inhibits RAD51, leading to the activation of the cGAS/STING/IFN cascade and DNA instability. Vitamin D prevents the loss of RAD51, thus limiting the adverse consequences of progerin [67]. Moreover, the shorter life expectancy associated with lower vitamin D levels could be related to telomere length. Clinical studies show that vitamin D level or intake is associated with longer leukocyte telomere length or telomerase activity [68]. In this context, DNA methylation at specific CpG dinucleotides has been proposed as a biomarker of biological aging [69]. Two human studies have shown that vitamin D levels or vitamin D supplementation are associated with lower epigenetic age and lower mortality risk [70,71].

Loss of proteostasis is another feature of aging [7]. Vitamin D supplementation promotes protein homeostasis and extends the lifespan of C. elegans by inducing the gene expression of Nrf2-like xenobiotic and oxidative stress-response factor (SKN-1) target genes [72]. Regulation of this pathway was also confirmed in mammals: in 1α(OH)ase-/- mice, 1,25D upregulated Nrf2, reduced ROS, decreased DNA damage, p16/Rb and p53/p21 signaling, cellular senescence and the SASP, and increased cell proliferation and lifespan [73].

Additionally, genetic defects in vitamin D pathway genes are associated with premature aging. VDR knockout (KO) mice, for instance, exhibit an aged phenotype [74]. VDR KO mice treated with a high-calcium, high-phosphate and high-lactose diet had a reduced life expectancy, smaller size, alopecia and changes in skin architecture [74]. VDR KO mice may also display altered insulin secretion [75], cardiac abnormalities [76], anomalous motor behavior [77] and other features resembling premature aging. 1α(OH)ase KO mice show a reduced lifespan, which can be reversed by exogenous 1,25D [73]. Interestingly, gene defects in vitamin D are associated with increased oxidative stress. In 1α(OH)ase KO mice, ROS levels were significantly increased in several tissues, and the expression of Superoxide dismutase 2 and Peroxiredoxin I were reduced in the skin and liver [73].

### 4.3. Vitamin D and the Immune System

**Figure 2:**
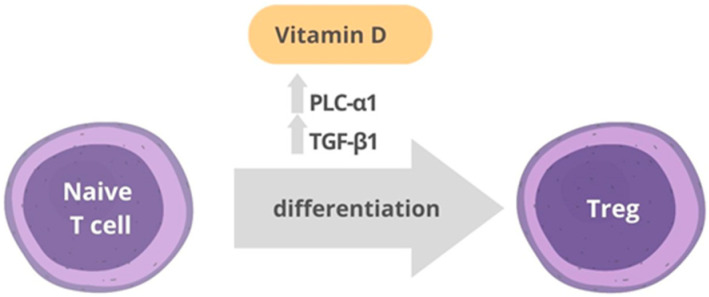
Vitamin D and T cells. 1,25(OH)2VD3 leads to an increase in the expression of PLC-γ1 and TGF-β1, ultimately resulting in the induction of FOXP3 expression and differentiation of naive T cells into regulatory T cells (Treg).

Among non-classic actions of vitamin D, anti-inflammatory effects may be relevant in modulating aging, as systemic inflammation is a predictor of mortality in the elderly [78,79]. 1,25D can regulate the immune system, decreasing inflammation and stimulating tolerance, as recently reviewed [10,11] Monocytes and macrophages can produce 1,25D, which enhances their antimicrobial activity via stimulation of cathelicidin LL-37 [80,81,82,83]. On the other hand, administration of vitamin D inhibited LPS-induced cytokine production in monocytes and macrophages via a mechanism mediated via upregulating MAPK phosphatase-1 and subsequent p38 and JNK inactivation [84]. Additionally, 1,25D inhibited NF-ĸB signaling through the upregulation of VDR target genes such as THBD [85] and LILRB4 [86]. Remarkably, locally activated 1,25D can also influence phenotype changes in different immune cell types. 1,25D inhibits lymphocyte T proliferation, modulates cytokine production [87,88] and induces regulatory T cells by modulating the VDR/PLC-γ1/TGF-β1 pathway [89] (Figure 2). VDR analogs suppressed Th17 differentiation in vitro by regulating STAT3 and RORgt, two key transcription factors regulating this process [90]. In addition, vitamin D modulates dendritic cell maturation and function [91]. 1,25D also stimulates the anti-inflammatory cytokine interleukin-10 in frail, pre-frail and fit patients [92].

### 4.4. Other Senescence-Related Targets of Vitamin D

Klotho is an anti-aging protein, mainly produced in the renal tubules [93]. Preclinical data showed that 1,25D upregulated Klotho gene transcription in the kidney [94]. In monocytes, Klotho prevented activation of the Golgi apparatus and endoplasmic reticulum stress response by upregulating CREB34L, TFE3, ATF6 and IRE1, inhibiting the gene expression of SASP components [95]. In this regard, Klotho also has anti-inflammatory effects through the regulation of pro- and anti-inflammatory factors, some of them components of the SASP, in culture cells and in vivo [96,97]. Klotho has shown to reduce oxidative stress in the cardiovascular system and vascular aging [98,99] and to modulate the inflammasome signaling pathway in pancreatic beta cells and in diabetic cardiomyopathy [100,101,102,103]. All these data support that Klotho regulation could be another potential mechanism involved in the anti-aging effects of vitamin D.

Sirtuins (SIRT) are a group of enzymes shown to modulate the senescence process [104,105]. These proteins rely on nicotinamide adenine dinucleotide (NAD+), which can modify proteins post-translationally [106]. Sirtuin-1 (SIRT1) is primarily located in the nucleus, regulating gene expression and deacetylating intracellular signaling proteins, including histones [107]. This protein is associated with longevity and has a negative regulatory effect on NF-κB signaling [108,109]. Furthermore, SIRT1 activation prevented endothelial senescence by deacetylating PGC-1α, activating PPARα and downregulating NADPH oxidase-mediated ROS production and NO inactivation [110]. Moreover, SIRT1 decelerated vascular calcification by modulating endothelial NO bioavailability and senescence, processes restored via vitamin D treatment [111]. In aging kidneys, SIRT1 expression was decreased and linked to changes in the expression of other target molecules, including PGC-1α/estrogen-related receptor-1α (ERR-1α), PPARα, Klotho and HIF-1α [112,113]. However, future studies are needed to demonstrate further the beneficial effects of targeting sirtuins on human senescence.

### 4.5. Vitamin D and CKD

**Figure 3:**
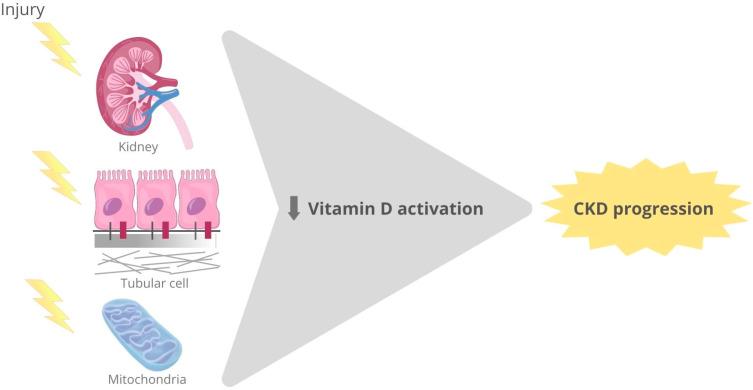
Vitamin D and the kidney. In response to injury, mitochondrial dysfunction in tubular epithelial cells diminishes 1,25D due to lower activation. Additionally, vitamin D deficiency contributes to kidney damage progression.

Endocrine 1,25(OH)2VD3 is mainly produced in the mitochondria of proximal tubular cells. In CKD, the damage of the proximal tubular cells limits the bioavailability of 1,25D due to a gradual decline in the kidney’s ability to produce 1α-hydroxylase, as aforementioned, thus contributing to the clinical manifestations of this disease (Figure 3). Loss of 1,25D production is a key contributor to the CKD mineral and bone disorder (CKD-MBD) that drives secondary hyperparathyroidism. Vitamin D repletion, calcitriol and VDR activators, such as paricalcitol, have long been used to prevent or treat secondary hyperparathyroidism. Additionally, more recent data have disclosed a function of VDR activation on kidney and cardiovascular health [44,45]. Serum 25-hydroxy-vitamin D [25(OH) D] levels between 20 and 30 ng/mL are considered insufficient and <20 ng/mL deficient [114]. Recent guidelines recommend measuring and treating vitamin D insufficiency in patients with CKD categories G3–G5 [115]. However, the active molecule and the dose to be used are still debated.

#### 4.5.1. Vitamin D Receptor Activation and Experimental Kidney Damage

In preclinical studies, treatment with vitamin D receptor agonists (VDRAs) displayed renoprotective actions, including anti-inflammatory effects [116,117]. One of the most studied VDRAs is the synthetic vitamin D analog paricalcitol (19-nor-1,25-hydroxyvitamin D(2)). In cyclosporine A-induced nephropathy, paricalcitol ameliorated renal dysfunction and reduced monocyte/macrophage infiltration, the over-expression of inflammatory cytokines within the kidney through the inhibition of mitogen-activated protein kinase signaling pathways [118,119]. On renal IRI in mice, paricalcitol reduced inflammatory cell infiltration and cytokine production via the upregulation of COX-2 and PGE2 [120]. In this model, inhibition of Toll-like receptor 4 (TLR4) activation has also been involved in paricalcitol anti-inflammatory effects [121]. In the unilateral uretheral obstruction (UUO) model in mice, paricalcitol diminished the infiltration of T cells and macrophages and the expression of RANTES and TNF-alpha in the obstructed kidneys [122]. Moreover, in experimental diabetic nephropathy induced via streptozotocin in rats, treatment with calcitriol or paricalcitol diminished total kidney mRNA expression of IL-6, monocyte chemoattractant protein (MCP)-1 and IL-18 without significant reductions in renal function or albuminuria [123]. In the focal segmental glomerulosclerosis mouse model induced via adriamycin, VDRAs decreased podocyte injury and proteinuria through the blockade of Wnt/β-catenin signaling [124]. 22-oxa-calcitriol decreased podocyte injury, limiting the effacement of foot processes and increasing nephrin, CD2AP and podocin expression [125]. Calcitriol reduced the expression of podocyte transient receptor potential cation channel C6 (TRPC6) [126]. Moreover, the combination of VDRAs plus the blockade of the renin–angiotensin system reduced albuminuria and podocyte damage [127]. In streptozotocin-induced diabetic nephropathy in mice, the concomitant administration of vitamin analog doxercalciferol and the AT1 antagonist losartan was the most effective intervention to reduce albuminuria, preserve the structure of the glomerular filtration barrier and diminish glomerulosclerosis [128]. Similar results were observed in diabetic Lprdb/db mice treated with losartan and paricalcitol [129]. All these preclinical data clearly demonstrate the anti-inflammatory actions of VDRAs in kidney damage.

**Figure 4:**
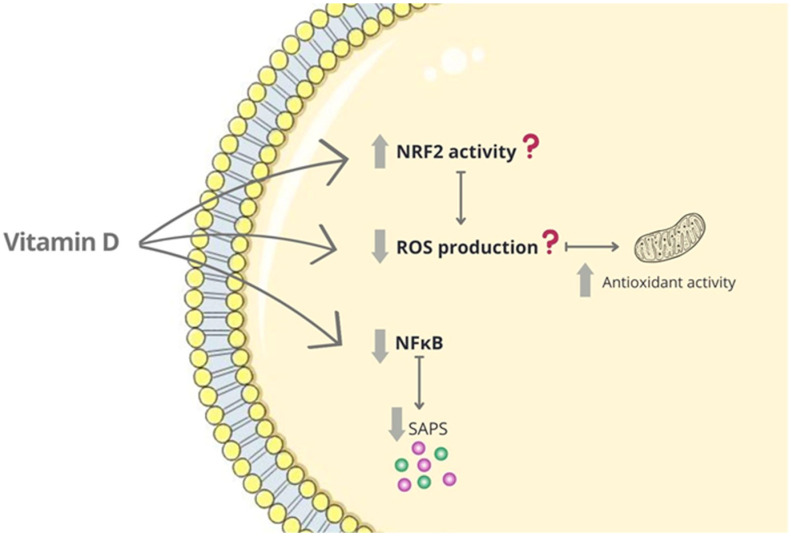
Vitamin D and cellular senescence in the kidney. Proposed mechanisms in response to injury; mitochondrial dysfunction in tubular epithelial cells diminishes 1,25D due to lower activation. Additionally, vitamin D deficiency contributes to kidney damage progression.

The NF-κB pathway is one of the most important signaling pathways involved in the regulation of inflammation and immune functions [130]. Some studies have proposed that paricalcitol can block NF-κB activation (Figure 4) [123]. Interestingly, in the UUO model, paricalcitol inhibits renal inflammatory infiltration and RANTES expression by promoting VDR-mediated sequestration of NF-κB signaling [122]. The activation of the canonical NF-κB1 pathway has been linked to pro-inflammatory gene regulation [122]. In particular, p65 phosphorylation on Ser536, subsequent translocation of the active p65–NF-κB complex to the nucleus and then binding to specific promoters have been proven to regulate gene transcription of pro-inflammatory genes, including MCP-1 and RANTES [121]. The activation of the noncanonical NF-κB2 pathway has also been recently related to kidney damage [66]. This pathway is regulated by TNF receptor-associated factors (TRAFs) and NF-κB–inducing kinase (NIK). In CKD patients, paricalcitol treatment restored circulating TRAF3 levels inhibiting the noncanonical NF-κB2 pathway and decreasing renal inflammation [66]. In IRI-induced AKI, paricalcitol reduced the inflammatory response by suppressing the TLR4-NF-κB signaling pathway and diminishing the expression of pro-inflammatory cytokines such as IL-6 and MCP-1 [131]. As commented before, in CKD, Klotho is downregulated. In a combined rat model of CKD with vitamin D deficiency, paricalcitol treatment did not change serum α-Klotho but ameliorated renal and vascular damage [132]. In experimental models of CKD, vitamin D-deficient diets induce renal fibrosis associated with TGF-β/Smad2/3-mediated EMT [133]. Studies in VDR gene-deficient mice have been conducted to assess the role of VDR in VDRA actions [66,134,135,136]. In UUO, renal lesions are exacerbated in VDR-deficient mice [137]. In this model, paricalcitol suppressed renal fibrotic markers such as TGF-beta1 [138]. Moreover, the administration of alpha-1-acid glycoprotein (AGP), a downstream molecule of calcitriol, attenuated fibrosis and inflammation in the UUO model [139]. Nrf2 is an important transcription factor involved in the antioxidant defense by the regulation of downstream gene expression of antioxidant enzymes, such as heme oxygenase-1 (HO-1). In a model of cisplatin-induced AKI, paricalcitol exerts a renoprotective effect by decreasing renal oxidative injury and inflammation through Nrf2/HO-1 signaling [140]. In this sense, SIRT1 has been shown to attenuate IR-induced kidney injury by activating antioxidant pathways such as Nrf2/HO-1 signaling [141]. SIRT1 may also provide protective effects against tubulointerstitial damage through the deacetylation of HIF-1α [113]. Moreover, the reduction in podocyte SIRT1 accelerates kidney injury [142]. Interestingly, few studies evaluate whether VDRAs could modulate senescence-related kidney damage. In this sense, in a model of contrast-induced AKI in rats, paricalcitol ameliorated kidney damage via the diminution of apoptotic cell death, mitophagy and downregulation of senescence markers p16 and **β**-galactosidase [143]. The absence of VDR in podocytes enhances the expression of p53, leading to increased transcription of Agt and AT1R, which activates the RAS pathway by downregulating SIRT1 [144]. As commented before, many components of the SASP are regulated by transcription factor NF-κB [33,34,35]. Therefore, the inhibitory effects on the activation of the NF-κB pathway described in response to paricalcitol in some studies suggest that paricalcitol could inhibit secondary senescence, although future studies are needed.

#### 4.5.2. Vitamin D Receptor Activation and Human Kidney Diseases

Many observational studies emphasize the association between 25(OH)D levels and cardiovascular disease, atherosclerosis, hypertension and heart disease in CKD patients and the elderly [145]. Vitamin D deficiency was an independent predictor of mortality in patients with or without heart failure [146,147,148,149]. It was suggested that seasonal fluctuations in mortality (i.e., decreased CVD-related deaths in the summer and increased deaths in the winter) might be related to the lower vitamin D levels in the blood during winter [150]. In observational and clinical studies, VDRAs were associated with lower cardiovascular and all-cause mortality rates, independent of the PTH level [20,151,152].

The clinical impact of vitamin D supplementation on kidney or cardiovascular disease beyond CKD-MBD has been a topic of debate among researchers. A review conducted by Chun Hu et al. [153] found that the evidence was insufficient to support the idea that vitamin D improves vascular function and reduces inflammation in such patients. On the other hand, Ding Dou et al. [154] believed that the benefits of vitamin D supplementation could vary depending on the dose and the progression of the disease. Meanwhile, Lundwall et al. [155] reported positive results from short-term vitamin D interventions in terms of improving endothelial function. However, two reviews [156,157] agreed that vitamin D supplementation does not positively affect cardiac function and structure. A recent meta-analysis also revealed that paricalcitol reduces the risk of cardiovascular events but does not protect renal function [157]. The different patient characteristics, including variables such as the prior existence of vitamin D deficiency, dietary salt intake, or other features, can explain the inconsistency of some data [158,159,160].

Beyond the impact on kidney or cardiovascular disease itself, vitamin D supplementation may improve certain pathologies associated with CKD. For example, a 2019 systematic review showed that normal or high levels of 25(OH)D and the use of supplements were associated with a lower risk of composite infection in patients undergoing long-term dialysis [161]. Another study published that same year showed that vitamin D treatment improved fasting glucose, HOMA-IR, triglycerides and cholesterol levels in CKD patients [162]. These findings could influence the possible association between vitamin D levels and mortality in patients with chronic kidney disease. In this regard, there is evidence that high vitamin D levels are associated with a lower risk of mortality from all causes in patients with chronic kidney disease [163], although other authors claim that the evidence is insufficient to support this [164].

## 5. Conclusions

Vitamin D deficiency is common in patients with CKD. Recent evidence supports that vitamin D or its analogs can provide pleiotropic protective effects on kidney injury. With the increasing prevalence of age-related diseases such as CKD, identifying novel treatment approaches for kidney disease and anti-aging therapies has become a significant focus of intensive research. However, current diagnostic and treatment tools are limited and marred by suboptimal markers for the identification of early damage or predicting disease progression difficulties. Understanding the underlying molecular mechanisms activated by vitamin D receptor agonists can shed light on the contradictory clinical findings and facilitate the design of novel therapeutic strategies. Additionally, cellular senescence mechanisms have been implicated in CKD and contribute to the natural aging process, further emphasizing the importance of studying novel treatment approaches for CKD and anti-aging therapies. Further investigation is needed to better understand the role of vitamin D in preventing and treating CKD and its effects on cellular senescence.

## 6. Future Perspectives

CKD research is focused on developing diagnostic and therapeutic tools to target the mechanisms of kidney injury. Research efforts are also focused on developing anti-aging therapies that target cellular senescence mechanisms and investigating the use of vitamin D. Studies should aim to understand the molecular mechanisms of vitamin D benefits in CKD and to develop better diagnostic tools to identify early damage. Better treatments are needed to improve patient outcomes and quality of life.

## Data Availability

Not applicable.
